# Comment on “Unclear Issues Regarding COVID-19”

**DOI:** 10.5152/eurasianjmed.2021.20322

**Published:** 2021-10

**Authors:** Sadaf Sheikh

**Affiliations:** Department of Emergency Medicine, Sultan Qaboos University Hospital, Muscat, Oman

## Dear Editor,

I read with great pleasure the review evaluating “unclear issues regarding COVID-19” by Yuksel et al.^1^ I found that there are certain unclear areas to be addressed such as cytokine storm and interleukin-6 inhibitor treatment in serious COVID-19 infections.

Coronaviruses present with an acute hyper-inflammatory syndrome, which activates dysregulated host immune responses. It initiates cytokine release syndrome featuring fever, hypoxia, and raised inflammatory markers. Key to this syndrome is the raised secretion of interleukin 6 (IL-6) by infected CD4^+^ T cells, macrophages, and dendritic cells. Viral load with raised IL-6 levels in critically ill COVID-19 patients is worth observing. Research into the IL-6 signaling may shed light on the evaluation of treatment options.

Experimental studies^2-^^4^ suggest that elevated IL-6 is found in severe infection; hence, cytokine storm blockers and immune host modulators have been used to deal with overwhelming inflammation. Cytokine storm with the release of IL-6, IL-1, IL-12, IL-18, and tumour necrosis factor-alpha (TNFα) was found in serious COVID-19 infections.^3,4^ These inflammatory mediators cause pulmonary inflammatory response with increased alveolar-capillary gas exchange causing oxygenation challenging in these patients. Hypoxia and the profound inflammatory response observed in COVID-19 pneumonia encouraged scientists to use IL-6 or IL-6 receptor-blocking antibodies. Agents such as Tocilizumab, Sarilumab, and Siltuximab are being used in clinical trials in severe COVID-19 pneumonia.^3,4^ Tocilizumab deals with soluble and membrane-bound IL-6R with complete IL-6 signaling blockade, resulting in rapid recovery from fever and respiratory distress. A non-peer-reviewed study^4,5^ was conducted on 21 patients with severe COVID-19 symptoms in which Lopinavir and Methylprednisolone with 400 mg of Tocilizumab single dose were given. Improvement with low requirement for oxygen, respiratory symptoms, and normalizing lymphocyte counts were observed. No adverse events or deaths were observed. Interleukin-7, another possible treatment modality, is getting importance in COVID-19 and sparks treatment hope.^6^

IL-6 is a pivotal cytokine and has attracted high levels of interest, and antibodies that block the IL-6 receptor (Tocilizumab and Sarilumab) are currently under phase 2/3 clinical trials for the potential treatment of COVID-19.^3,4^ Fewer agents dealing with TNF-blocking antibodies such as Adalimumab, Etanercept, and Golimumab have been recommended now in severe COVID-19 illness.^3,4^

Not all patients respond to TNF or IL-6 inhibition, and this could be explained by neutralizing antibodies that developed against the blocking agent. Increased susceptibility with bacterial infections is another pressing concern due to impaired acute phase response due to IL-6 blockade. Anti-IL-6 therapy is designed with tailored function toward specific disease. It is imperative for physicians to evaluate a cytokine panel to identify the needs of each patient before administration of selective immunosuppressive therapy. A combination of antiviral and immunosuppressive therapies that decrease the viral load should be considered for future treatment options.
